# Short-term epileptic seizures prediction based on cepstrum analysis and signal morphology

**DOI:** 10.1186/s42490-024-00081-1

**Published:** 2024-07-01

**Authors:** Bahar Tajadini, Saeid R. Seydnejad, Soheila Rezakhani

**Affiliations:** 1 Department of Electrical Engineering, Shahid Bahonar University of Kerman, Kerman, Iran; 2 Department of Neurology, Neuroscience Research Center, School of Medicine, Kerman University of Medical Sciences, Kerman, Iran

**Keywords:** Epileptic seizure prediction, Wavelet transform, Autoregression model, Cepstrum, Adult epileptic model

## Abstract

This article aims to provide and implement a patient-specific seizure (for Intervention Time (IT) detection) prediction algorithm using non-invasive data to develop warning devices to prevent further patient injury and reduce stress. Employing algorithms with high initial data volume and computations time to increase the accuracy is an important problem in prediction issues. Consequently, reduction of calculations is met by applying only two effective EEG signal channels without manual removal of artifacts by visual inspection as the algorithm’s input. Autoregression (AR) modeling and Cepstrum detect changes due to IT period. We carry out the goal of higher accuracy by increasing sensitivity to interictal epileptiform discharges or artifacts and reduce errors caused by them, taking advantage of the discrete wavelet transform and the comparison of two channels epochs by applying the median filter. Averaging and positive envelope methods are introduced to patient-specific thresholds become more differentiated as soon as possible and can be lead to sooner prediction. We examined this method on a mathematical model of adult epilepsy as well as on 10 patients with EEG data. The results of our experiments confirm that performance of the proposed approach in accuracy and average false prediction rate is superior to other algorithms. Simulation results have been shown the robustness of our proposed method to artifacts and errors, which is a step towards the development of real-time alarm devices by non-invasive techniques.

## Introduction

EPILEPSY is a chronic disorder and outburst of the brain function that is caused by abnormal electrical discharge. Its symptoms are sudden seizures, temporary anesthesia, and intermittent seizures [1, 2]. More than 50 million people worldwide have epilepsy [2] that suffer from injuries due to the loss of consciousness or delayed response to the onset of seizures.

Therefore, due to the importance of reducing injuries many studies have addressed the prediction of epileptic seizures by analysis of Electroencephalogram (EEG) recordings. According to the Assi study, epileptic EEG is divided into 5 states that Intervention Time (IT) state occurs a few seconds before the onset of ictal [3].

The seizures detection is also discussed in addition to predicting seizures in epilepsy. The purpose of detection is to distinguish ictal period but detection the pre-ictal period to create warnings for medical procedures that are determined hours or minutes before ictal is the aim of prediction and do not necessarily mean the exact time of ictal [4].

Prediction algorithms can be classified into two broad categories: short-term and long-term [3]. Therapeutic operations include reducing the frequency of seizures or preventing the occurrence of seizures with drugs or nerve stimulation, or preventing traumatic events to the patient [4]. For this reason, short-term prediction can also help us in this direction. The IT period must also be identified from interictal as there is no specific time for IT.

Numerous studies have been performed to detect and predict seizures. Luders considers the detection algorithms in three categories of 1) Pre-classification, 2) State and 3) Trending [4]. Pre-classification algorithms employ EEG data in training phase. This scheme classified into two categories: seizure and non-seizure [4].

State algorithms measure the absolute state of the current epoch and compare it with the non-seizure state previously defined or obtained from previous data [4].

Some algorithms use modeling methods to extract features. Toward this, AR approach [5, 6], AR Moving Average [7], dynamic model with hidden variables [8] and the sparse Laguerre Volterra AR [9] have investigated in literature. Other methods used in the literature include the Lag Synchronization Index to compare phase synchronization between chaotic oscillators [10], the largest Lyapunov exponent [11], Empirical Mode Decomposition and wavelet transform [2], decorrelation time, power spectrum, Spectral Edge Frequency, criteria based on entropy and probability [12].

The database used in most studies has already been preprocessed by removing artifacts by visual inspection. While EEG is contaminated by natural environment artifacts, the prediction algorithm should be able to have good performance in the presence of artifacts. In [13, 14], the Cepstrum method is proposed to diagnose seizures, to be used on EEG and Intracranial EEG (IEEG) multi-channel separately in short term, whose artifacts have been removed and which includes different patient states. According to the hypothesis [15], the nonlinear problem of EEG caused by signal convolution is solved by Mel-frequency Cepstrum for improving the detection efficiency. Method of [14] was improved in 2014 by calculating the signals Teager energy Cepstrum and then following the same procedure as before[13].

Trending algorithms are to compare the current epoch with the background section. The background epoch is defined as a data window before the current epoch, which also changes over time. The features extracted from the two epochs are compared with the classification or threshold method. By determining the current and background epochs, Gotman makes a diagnosis by comparing the average half-wave amplitude, the coefficient of variation, and average half-wave duration features of the current and background periods, then thresholding between them [16, 17].

So far, we reviewed the studies which have been based on long-term prediction. To the best of our knowledge, only few studies have been performed on short-term prediction. Following this purpose, Osorio’s method [18] was presented using trending algorithms in two steps, Finite Impulse Response (FIR) filter and median filter over all IEEG data channels. Decomposition of signals into seizure and nonseizure and increasing the specificity of seizure detection process to artifacts are performed by FIR filter and median filter respectively. Processing all channels at steps increases computational time, which can be reduced by reducing the number of channels to focal channels in focal patients. According to the results of comparison to [18]’s method in this study, it can be understood that the median filter alone cannot adequately increase the specificity of seizure detection from Interictal Epileptiform Discharges (IEDs) and artifacts, so there is a need to add other steps. Also, artifacts and noise have a greater effect on EEG than IEEG [12, 19]. However, seizure warning methods that have clinical application are generally based on the use of non-invasive EEG [10], and it is also easier to access EEG. In 2019, a patient-specific study was conducted based on finding synchronization patterns. In the threshold-based classification approach used in this study, a single feature and a single threshold must first be obtained, which requires setting the unknown parameters of the algorithm. In each run, which is applied to two sets of features computed over the two synchronization measures Phase Lag Index (PLI) and Weighted PLI (WPLI), and considered in three different lengths of time, parameters must be set for each patient. Cases that have better performance are considered. This algorithm evaluates all data channels as in the previous study. Also, the main challenge of this method is to tune a large number of its parameters [20].

We study the short-term seizure prediction algorithm motivated by the acceptable accuracy and specificity of forecast and considering that to the best of our knowledge, there is no high accuracy algorithm with low computational cost; we aim to accept this challenge and progress an effective method. Moreover, we take advantage of methods that are sensitive to regular and slow periodic changes, which can detect the IT period of the interictal. Also, an efficient prediction algorithm to increase accuracy must attenuate artifact and IEDs. This issue is discussed thoroughly in this article. It can be seen that with more data processing steps, the prediction performance improves. Let us now summarize our main contributions based on three aims as follows.

1. The IT period detection problem has been satisfied using AR modeling and Cepstrum Analysis.
2. The challenges of enhancing the accuracy and reducing the False-Positive Rate (FPR) are solved by the Discrete Wavelet Transform (DWT) and comparing the current epoch and background section by exerted median-filter.
3. Finally, to prepare the processed EEG for thresholding, averaging and positive envelope approaches are utilized. The performance of the proposed algorithm is inspected with two other approaches, EEG data from hospitalized patients and the proposed synthetic EEG.

The rest of this article is organized as follows. Section II introduces the proposed algorithm for IT detection giving a full discussion of the motivation for utilizing disparate steps. How to generate the synthetic EEG of the EEG Adult model is described in Section III. Section IV is allocated to the presentation and assessment of our simulation results. Finally, Section V concludes this article.

*Notations:* We use capital, bold small, and small letters to represent a matrix, a vector, and a scalar variable, respectively. The symbols e, T, and log are the exponential, transpose, and logarithm functions, respectively and the k variable represents the sample number of signal.

## Methodology

We formulated the seizure prediction issue as a thresholding task between interictal and IT periods, so the main problem is determining the optimum threshold value that is able to recognize IT faster with less error. Based on the quasi-periodicity of IT, we came up with the idea to relax the recognition problem by employing approaches that are periodicity sensitive and applicable to biological signals. Certainly, the recognition of quasi-periodic periods alone may not be accurate enough, and if we know how to incorporate time frequency approaches effectively, the algorithm’s sensitivity can be further extended. If we made more profit of dissimilarity of EEG temporal lobes channels over time, it could be much closer to less error. So, to tackle the thresholding problem, the following three subproblems should be considered. 1) what periodicity sensitive approaches can be benefited for EEG? 2) If periodicity recognition was acceptable, how to increase accuracy by embedding time–frequency approaches? In order to improve the prediction performance, how to take advantage of the different channels’ changes of EEG over time?

To sum up, our design of the short-term prediction algorithm consists of three main stages: preprocessing, seizure prediction, and signal preparation to determine thresholds. Each stage contains steps to solve the sub-problems stated above. The procedure of our algorithm starts with the preprocessing stage to detrend and limit the frequency band by filtering on two EEG channels. Then the seizure prediction stage is executed. In this stage first, preprocessed signals are time–frequency decomposed by DWT method then, weak periodicities are detected by AR-Cepstrum approach and finally the current epochs are compared to background sections of channels by applying a median filter. The procedure ends by calculating an average and obtaining a positive envelope of processed signal for more accurate detection of the IT’s onset.

### Preprocessing

For this research, we used segmented recordings from two temporal lobe channels, without the manual removal of artifacts by visual inspection are normalized by elimination of linear trend then bandpass filter is applied to more benefit their effective epileptic frequencies. Beforehand, it is important to choose the proper window length for segmentation. Some information will be lost and the real-time target will not be considered by large window length and the small window caused an unnecessary increase of calculations and a loss of time. By trying several different lengths, as a result, we have chosen a length of 5 s (1375 samples) with 80% overlap. The FIR band-pass filter with a Hamming window is selected for the linearity of the filter phase. To apply a band-pass filter, despite the importance of how to determining the bandwidth and the order of the filter, due to the large volume of its description, we are satisfied with its final values. The bandwidth and order were selected [6 − 20] Hz and 220 respectively. We analyzed the orders of 80, 220, and 500 for the 6 to 20 Hz range to determine the appropriate filter’s order. For this purpose, part of the training data was considered for seizure detection using pre-processing, AR modeling and Cepstrum steps. According to the results of the detection accuracy, it can be concluded that the 220 order filter preserves more morphological information for seizure detection than the other two filters.

### Seizure prediction

***Step 1. Discrete wavelet transform:*** As the first step of this stage, the DWT is superior to the frequency transform methods for seizure detection because it provides almost simultaneous time–frequency signal information at multiple levels of resolution, which apply to the two preprocessed signals. The reason for the higher resolution is the use of windows with variable lengths. DWT $$(x[]$$.$$)$$ corresponds to the decomposition of $$x[.]$$ into vectors of different resolutions, with the scaling and wavelet functions being used as the corresponding basis. Thus, the signal in each step is decomposed into two parts: low frequency (approximation signal) and high frequency (detail signal). The DWT is defined as [21]:

1 $$c\left({j}_{0},k,ch\right) =\frac{1}{\sqrt{M}}\sum_{n} x(n,ch){\phi }_{{j}_{0},k}(n)$$

2 $$d(j,k,ch) =\frac{1}{\sqrt{M}}\sum_{n} x(n,ch){\psi }_{j,k}(n)$$

where $x(n,ch)$ is certain discrete signal channels, $\psi$ is the mother wavelet function, $\phi$ is the scaling function, $d\left(j,k,ch\right)$ is the detail coefficients, and $c\left({j}_{0},k,ch\right)$ is the approximation coefficients, and the signal is represented on these coefficients. Normally we let ${j}_{0}=0$ and select M to be a power of 2 (i.e., $M={2}^{J}$) so that the summations in Eqs. 1 and 2 are performed over $n=\mathrm{0,1},2,M-1$, $ch=\mathrm{1,2}$, $j=\mathrm{0,1},2,,J-1$ (scale or level parameter), $k=\mathrm{0,1},2,,{2}^{j}-1$ (shift parameter). The Daubechies (db) 4 and 6-levels $(J-1=6)$ of resolution were chosen for use in this step. Daubechies is computationally appropriate, because it’s power spectral density estimates at different levels of resolution can distinguish and decompose common interictal and seizure frequency bands, and its impulse response is like an epileptiform discharge. According to the results obtained on several patients, we realized that the detail coefficients were more effective in IT detection, therefore we utilize 12 detail coefficients signals obtained from two channels data and Eq. 2 as the [det] parameter in Algorithm1.

*Step2. Autoregression model:* Signal parameterization of partial coefficients segmented by the AR model is an important part of this algorithm that helps to detect IT period of weak periodicity. This model is a versatile mathematical model used to display the rather irregular and non-stationary EEG signal, also has been the most widely used method for predicting/ detecting seizures among other modeling methods. Therefore, if ${x}_{k}$ is considered as EEG time series, the regressors vector of the model is ${\phi }_{k}\triangleq {\left[{x}_{k-1},{x}_{k-2},\dots ,{x}_{k-n}\right]}^{\mathrm{^{\prime}}}$, the model coefficients vector is $\mathbf{a}\triangleq {\left[{a}_{1},{a}_{2},\dots ,{a}_{n}\right]}^{\mathrm{^{\prime}}}$ and the AR model is expressed as Eq. (3) [5, 22]:

3 $$\begin{array}{c}{x}_{k}={\left({\phi }_{k}\right)}^{T}{{\varvec{a}}}_{k}+{e}_{k}\end{array}$$

where $n$ is the model order, ${e}_{k}$ is white noise error signal with zero mean and ${a}_{j}(j=\mathrm{1,2},\dots ,n)$ are unknown parameter vectors which are estimated with recursive online estimators. The proposed method has the potential to be implemented in real-time brain implant systems. In the Recursive Least Squares (RLS) method, the AR coefficients are estimated in such a way to minimize the cost function $(J)$:

4 $$\begin{array}{c}J\left({\widehat{{\varvec{a}}}}_{k}\right)=arg\ \underset{{\widehat{{\varvec{a}}}}_{k}}{{\text{min}}} \sum\limits_{i=1}^{k} {\lambda }^{k-i}{\epsilon }^{2}(i,\varvec{a}), 0<\lambda <1\end{array}$$

where the ${\widehat{{\varvec{a}}}}_{k}$ estimates ${{\varvec{a}}}_{k},\lambda$ is the forgetting factor, and $\epsilon \left(i,{\varvec{a}}\right)$ is prediction error obtained from Eq. (5):

5 $$\begin{array}{c}\epsilon \left(k,\widehat{{\varvec{a}}}\right)={x}_{k}-{\left({\phi }_{k}\right)}^{T}{\widehat{{\varvec{a}}}}_{k-1}\end{array}$$

and the coefficients ${{\varvec{a}}}_{k}$ are recursively estimated as follows:

6 $${\widehat{\varvec{a}}}_k={\widehat{\varvec{a}}}_{k-1}\;+\;\varvec{k}_{k}{\epsilon}_k$$

here, ${\widehat{x}}_{k}$ is the estimate ${{\varvec{a}}}_{k}, {\widehat{{\varvec{a}}}}_{k}$ estimates ${{\varvec{a}}}_{k}$, and ${{\varvec{k}}}_{k}$ is gain in sample $k$ with dimensions $n\times 1$:

7 $$\begin{array}{c}{{\varvec{k}}}_{k}={{\varvec{P}}}_{k}{{\varvec{\phi}}}_{k}\end{array}$$

and ${{\varvec{P}}}_{k}$ is the inverse correlation matrix of the model coefficients with dimensions $n\times n$ obtained by Eq. (8):

8 $$\begin{array}{c}{{\varvec{P}}}_{k}=\left({{\varvec{P}}}_{k-1}-\frac{{{\varvec{P}}}_{k-1}{{\varvec{\phi}}}_{k}{{\varvec{\phi}}}_{k}^{T}{{\varvec{P}}}_{k-1}}{\lambda +{{\varvec{\phi}}}_{k}^{T}{{\varvec{P}}}_{k-1}{{\varvec{\phi}}}_{k}}\right)\frac{1}{\lambda }\end{array}$$

The estimated ${x}_{k}$ is obtained from Eq. (9):

9 $$\begin{array}{c}{\widehat{x}}_{k}={\left({\phi }_{k}\right)}^{T}{\widehat{{\varvec{a}}}}_{k-1}\end{array}$$

Usually, the initial values and the vector of coefficients are considered ${{\varvec{P}}}_{0}=\delta {\varvec{I}}$ and ${{\varvec{a}}}_{0}=[0]$, where $0<\delta <1$ and I are the identity matrix. The outputs of previous step are first windowed by length of 500 samples and 75% overlap, which is introduced as the [seg] parameter in Algorithm1. The forgetting factor is set to 0.99, and the appropriate order of the AR model is found using the model order estimation criteria, such as Akaike Information Criterion (AIC), Final Prediction Error (FPE), and cost function, mentioned in reference [23]. According to these criteria, the most suitable order is 8, and then the AR model is recursively estimated (Eq. (9)) and creates the [est] parameter in Algorithm1. The length and number of the estimated model will be equal to the length and number of outputs of the previous step. Step 3. Cepstrum: The inputs are segmented with a window as before and Cepstrum is applied on them, represented by the [ceps] parameter in Algorithm1. The real Cepstrum is defined as the inverse Fourier transform of the log magnitude spectrum. First, the Discrete Fourier Transform (DFT) signal is obtained as follows[13].

10 $$\begin{array}{c}X(k)=\frac{1}{N}\sum\limits_{n=1}^{N} x(n){e}^{-j\left(2\pi \frac{k}{N}\right)n}\end{array}$$

The Cepstrum coefficients of the discrete signal is expressed as Eq. (11).

11 $$\begin{array}{c}C(n)=\frac{1}{N}\sum\limits_{k=1}^{N} log(|X(k)|){e}^{j\left(2\pi \frac{k}{N}\right)n}\end{array}$$

According to the hypothesis of [15], EEG signals can be regarded as the result of the convolution of two functions generated by motivation signals and the common effect of the neuronal structures. This reference, proposes the deconvolution method for separating the EEG from different components. Therefore, the use of cepstrum converts the convoluted signal to a time-domain linear signal. Other reasons for choosing this method are mentioned below.

1. Cepstrum is used to detect unvoiced from voiced speech that has slow and periodic changes. The seizure of the EEG signal has a regular and periodic rhythm compared to the interictal period, and the Cepstrum can be used to detect seizure’s onset. 2. Moreover, a limited number of Cepstrum coefficients can be considered in signals, such as EEG, with a long length because the final Cepstrum coefficients are close to zero. According to the obtained results, the first coefficient is sufficient and appropriate for prediction. Therefore, we utilize the first coefficient by Cepstrum calculation. As a result, the number of outputs of this step is the same as the previous steps [15, 24].

***Step 4. Median filter and comparing the background series with the current series:*** Wavelet and median filter steps are used to improve the seizure detection process of certain artifacts, interictal epileptiform discharges, or even normal activities such as alpha waves following the second aim. The median filter is able to remove random noises and can somewhat preserve image details and image edge. This property makes it a good choice for accurate detection of the “edge” between the seizure’s onset and non-seizure states.

The EEG signal contains both positive and negative values, and signal magnitude was higher in the ictal period than in the interictal period. To increase the difference between the two periods, we first obtain its square and then pass the squared data through the median filter with a moving window of a certain length. The recent current period $\left(F{G}_{k}\right)$ is obtained from Eq. (12) [18].

12 $$\begin{array}{c}F{G}_{k}=median\left\{{{\text{y}}}_{{\text{k}}}^{2},{{\text{y}}}_{{\text{k}}-1}^{2},\dots ,{{\text{y}}}_{{\text{k}}-{\text{q}}+1}^{2}\right\}\end{array}$$

Here, $q$ is window length or filter order and equal to one second, which is determined according to the sampling rate. Using this EEG feature, which has a different morphology at different times and channels, we can increase accuracy and reduce error. Therefore, the changes of the current period are measured compared to the background period of two channels. The background period is obtained by passing the current period through the median filter and then “exponentially forgetting” the output. The forgetting factor is a parameter used in adaptive filtering. It provides a way to control the influence of older observations on the current estimate. Adjusting the “forgetting factor” $\left(\lambda \right)$ allows to set the length of the window corresponding to the background to any length from seconds to days, without increasing the computational burden. This, combined with the median filter insensitivity to spurious signal changes, allows accurate representation of the history of the signal and timely adaptation to state changes. The background period is obtained from Eq. (13).

13 $$B{G}_{k}=\left\{\begin{array}{ll}\left(1-\lambda \right)\text{ median }\left\{{{\text{FG}}}_{{\text{k}}},\right.& \\ \left.F{G}_{k-s},\dots ,F{G}_{k-\left({q}_{2}-1\right)s}\right\}+& \\ \left(\lambda \right)B{G}_{k-1}& k=ns\\ B{G}_{k-1}& n\left(s-1\right)\le k<ns\end{array}\right.$$

where $n=\mathrm{0,1},2,\dots ,s=0.5\mathrm{ sec}$ is half the sampling rate, ${q}_{2}$ is equal to the sampling rate, and $\lambda =0.99$ is the forgetting coefficient. The dimensionless ratios for each channel are calculated from Eq. (14).

14 $$\begin{array}{c}{r}_{k}^{(j)}=\frac{F{G}_{k}^{(i=1)}}{B{G}_{k}^{(j)}}\end{array}$$

Here, $i=1$ (main channel), $j=\mathrm{1,2}$ and $j=2$ (non-main channel) are the channels number. The median filtering method and the generation of the background period were tested on a patient’s EEG, indicating the ability to identify seizure’s onset and non-seizure edges [18]. At the input of this step, we have 12 signals, 6 of which are related to the main channel, and the other 6 are related to the non-main channel. This step has two parts according to Eq. (14): the first part is the ratio of the first channel to itself, and the second part is the ratio of the first channel to the second channel, so we will have 12 outputs which are the same as [rat] parameter in Algorithm 1.

**Figure:**
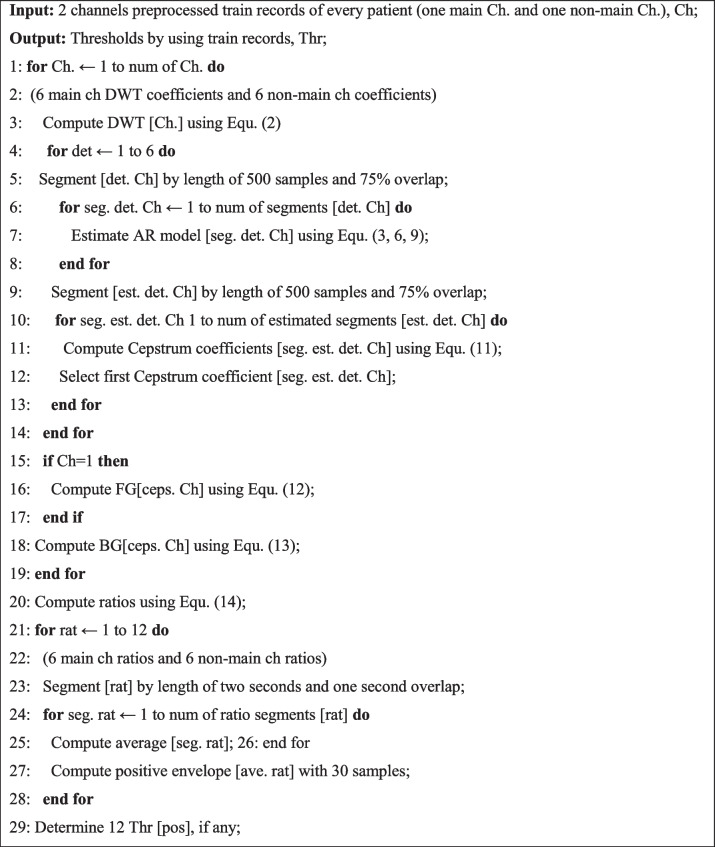
**Algorithm 1.** EEG training prediction algorithm

### Preparation to determine the thresholds

**Fig. 1:**
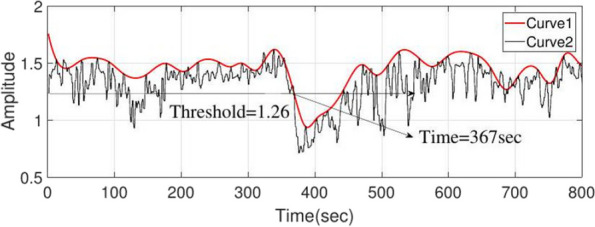
Curve 2 shows the average output of the series comparison stage using the leading window, and curve 1 is the envelope curve of curve 2

*Averaging and evelope of the curve:* The third stage of the algorithm, which includes averaging and envelope steps, are suitably designed with the following three objectives: 1. To eliminate bursts and high-frequency artifacts, 2. To down sample and 3. To smooth the achieved signal. Given that the averaging method eliminates the initial changes of the IT period, we consider the length of the window in two seconds with one second overlap ([ave] parameter in Algorithm 1). In Fig. 1, curve 2 is the average and the start of the IT period occurs in 360 s, which cannot be determined on curve 2 by the threshold method. The calculation of the positive envelope ([pos] parameter in Algorithm 1) of curve 2 will yield curve 1, which can facilitate the threshold determination of the start of the IT period. The variable that must be specified in this step is the number of samples between the two peaks of the curve, and a value of 30 was appropriate here.

***Thresholding:*** The results obtained from the positive envelope curves indicate different thresholds for predicting the seizure of each patient; thus, a different threshold is considered for each patient. According to the 12 output signals, the threshold values are selected with the least error and the most appropriate delay from the start of the seizure. The proposed training and test algorithms are summarized in Algorithms 1 and 2, respectively, and how to apply the thresholds to the new data in more detail and evaluate the performance of the algorithm are given in the IV-B section.

**Figure:**
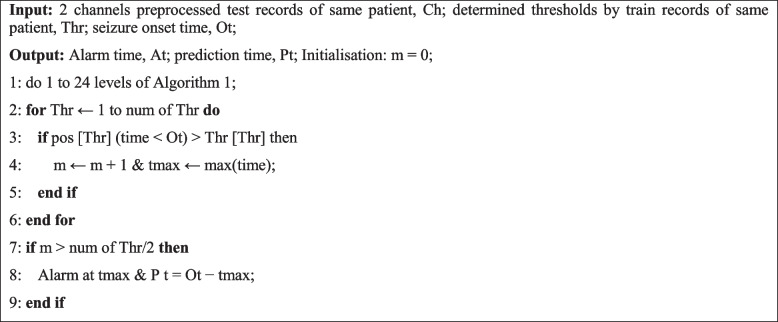
**Algorithm 2.**  EEG test prediction algorithm

## Adult epileptic eeg signal model

The structure of the Celka model which was formerly proposed based on Non-Gaussian and non-statistic EEG by Roessgen and Lopesdasilva [25–27], has since been used by other researchers to detect seizures in infants. In this study, the proposed model includes ictal, interictal, and IT periods, which are derived from the Celka model and present adult dataset. In adults, seizures typically occur at a frequency between 0.5 and 10 Hz. However, for newborns and infants, seizures tend to have a lower frequency content. To date, no model of adult epileptic EEG has been proposed that is suitable for the prediction. To cover the differences between the neonatal and adult EEGs to some extent, we change the frequencies of Celka model and determine the filter coefficients used in this model by using the real adult data available.

**Fig. 2:**
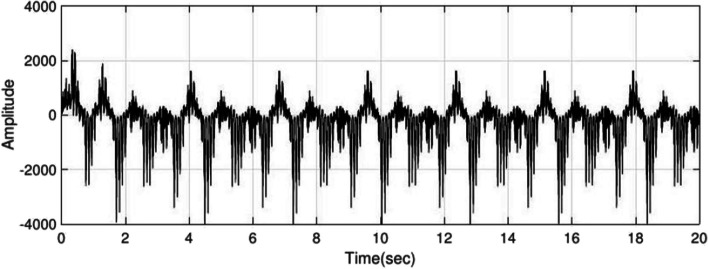
Inter-ictal period EEG obtained from the adult epileptic model

**Fig. 3:**
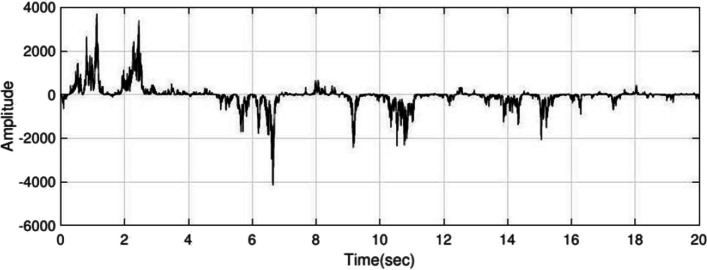
Ictal period EEG obtained from the adult epileptic model

**Fig. 4:**
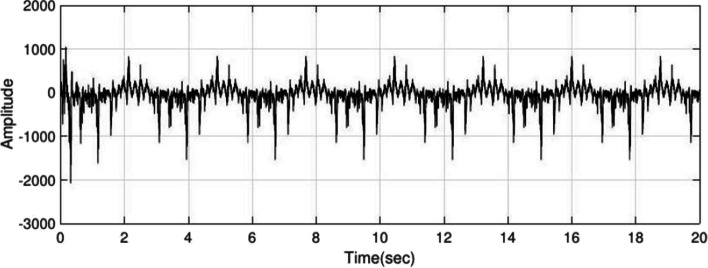
IT period obtained from the adult epileptic model

Using the interictal period of the present adult dataset, ${H}_{1}(z)$ and ${H}_{2}(z)$ are estimated with order $=2$ and to produce epileptic EEG, set the ${f}_{m}=1.5 \, {\text{Hz}}$ in equation $S(k)$. Examples of interictal and ictal are given in Fig. 2 and 3, respectively. The IT period has less amplitude and frequency than ictal, so by setting the frequency ${f}_{m}$ to 1 Hz and lower amplitude is produced as ictal, of which Fig. 4 is an example.

### Simulation and results

#### Data collection

Data were collected from EEG of patients with temporal lobe epilepsy through Long-Term Monitoring (LTM) by the authors in Mehrgan Hospital in Kerman, the largest East province of Iran for diagnostic purposes only, which was also used in this study. In this study, 26 electrodes with a sampling rate of 256 Hz, 11 h of interictal data, and 34 min of ictal data were performed with an international 10–20 system of EEG electrode positions on the EEG of 10 patients. Data were collected while the hospitalized patients were awake and asleep. Details of the patients’ EEG of dataset and data employed for generating synthetic adult EEG, such as age, gender, type of epilepsy, localization, number of seizures, amount of InterIctal, and Ictal data training and testing are given in Table 1. There are several records for each patient, some of which are randomly considered as training data. According to researches, the accuracy of predicting multi-channel epileptic seizures is better than one channel [3], according to Temporal Lobe Epilepsy (TLE) patients, two channels are selected from the temporal lobes. The main channel is the seizure focus (main) and the second channel is selected opposite the seizure focus (non-main). There are many artifacts on the data due to their long duration and the patient’s daily activities. In the proposed algorithm, the intervals with artifacts are not removed and the data are processed completely and continuously, yielding acceptable results. Data are analyzed with MATLAB R2017b software. It should be noted that the purpose of this study is not to locate the focus of seizures, and the channel is selected non-automatically and the selected channels are also fixed in different records.

**Table 1:** Information of EEG data

Pat. n	Age (years)	Gender	Type of epilepsy	Localization	#Sei. Tr	Int.Tr (sec)	Int. Tr (sec)	#Sei.Te	Int. Te (sec)	Int. Te (sec)
1	32	Female	TLE		1	677	73	-	800	-
2	38	Male	TLE		2	1744	91	4	3759	182
3	29	Male			3	2657	184	2	5955	192
4	18	Male	TLE	Left temporal	1	1500	130	1	3707	91
5	32	Female	FLE	Left frontal	1	1435	65	-	2195	-
6	30	Female	TLE	Right temporal	1	740	64	-	1200	-
7	32	Male	FLE	Left frontal	1	1255	72	1	3350	100
8	21	Male	TLE		1	2641	108	3	3367	308
9	38	Female	TLE	Right temporal	1	210	90	-	350	-
10	25	Female	TLE		1	1900	100	2	1620	200
	-	-	-	-	2	5780	200	3	2015	600

*Pat. n *patients number,* #Sei.Tr* #Seizure Train, *Int.Tr* Interictal Train, *Ict.Tr* Ictal Train, *#Sei.Te* #Seizure Test, *Int.Te* Interictal Test, *Ict.Te* Ictal Test

### Thresholding and performance evaluation of the proposed method

**Fig. 5:**
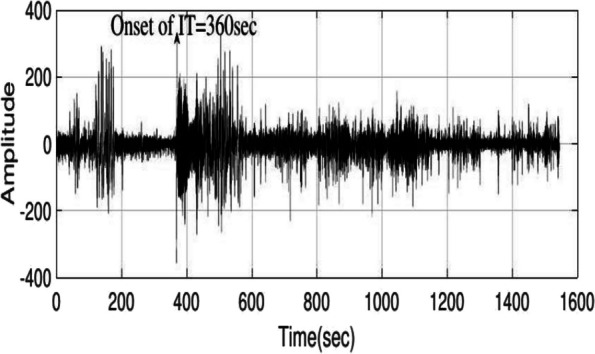
Signal of a patient without processing at the start of IT period in 360 s

**Fig. 6:**
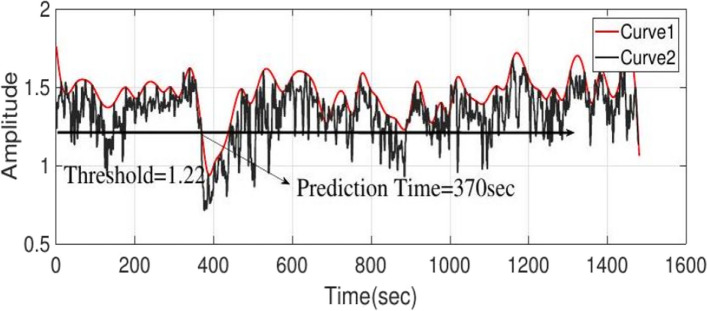
Curve 1 is the first output, which is the result of comparing the first channel to itself resulting from the first detail coefficient in the wavelet; curve 1 is the envelope curve of curve 2; the threshold value is 1.22 and the prediction time is 370 s

**Fig. 7:**
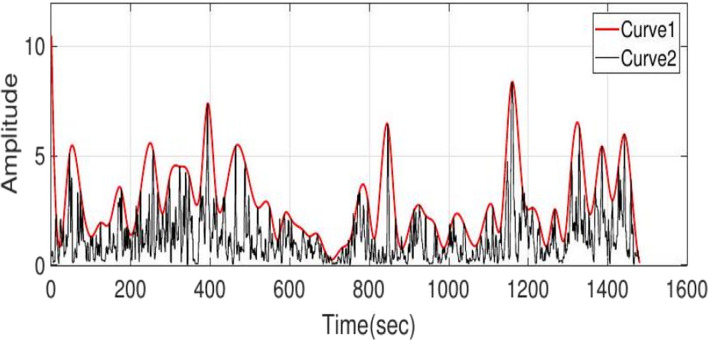
Curve 1 is the seventh output, resulting from the comparison of the first channel with the second channel obtained from the first detail coefficient in the wavelet; curve 1 is the envelope curve of curve 2, which is not able to recognize the IT period and is not considered here

In prediction, the thresholding is done according to 12 output signals of positive envelop step. First, the thresholds for each signal are specified according to the training data. Some signals may not have a low-error threshold for prediction; thus, this signal is not accepted for training. As a sample the results of a patient’s prediction are present in Fig. 5, showing an unprocessed EEG signal where the IT period occurs at 360 s. As mentioned above, we will have 12 outputs. Figure 6 shows the first of 12 outputs signal, which is a comparison of the main channel with itself and results from the first detail coefficient in the wavelet step. Curve 1 is the positive envelope of curve 2, and the threshold value is determined based on curve 1. The threshold value is assumed to be 1.22 to avoid error, and the prediction time is 370 s. Figure 7 illustrates the seventh output signal, resulting from the comparison of the main channel with the opposite channel due to the first detail coefficient in the wavelet step, and curve 1 is the positive envelope of curve 2. According to the figure, this output does not detect the IT period, thus, this output is not used for the test data in this patient.

Other acceptable thresholds are determined in the same way and examined in the test data. Given that the average length of seizure periods is usually 100 s, test records can be fractioned into 100-s intervals, which is the Seizure Occurrence Period (SOP) interval. The IT period is considered when half the outputs amplitudes of the intended interval of test data exceed their acceptable thresholds. For example, if there are seven out of 12 signals with an approved threshold in the training data, the amplitude of four signals must exceed the thresholds. This process is performed for each patient.

**Fig. 8:**
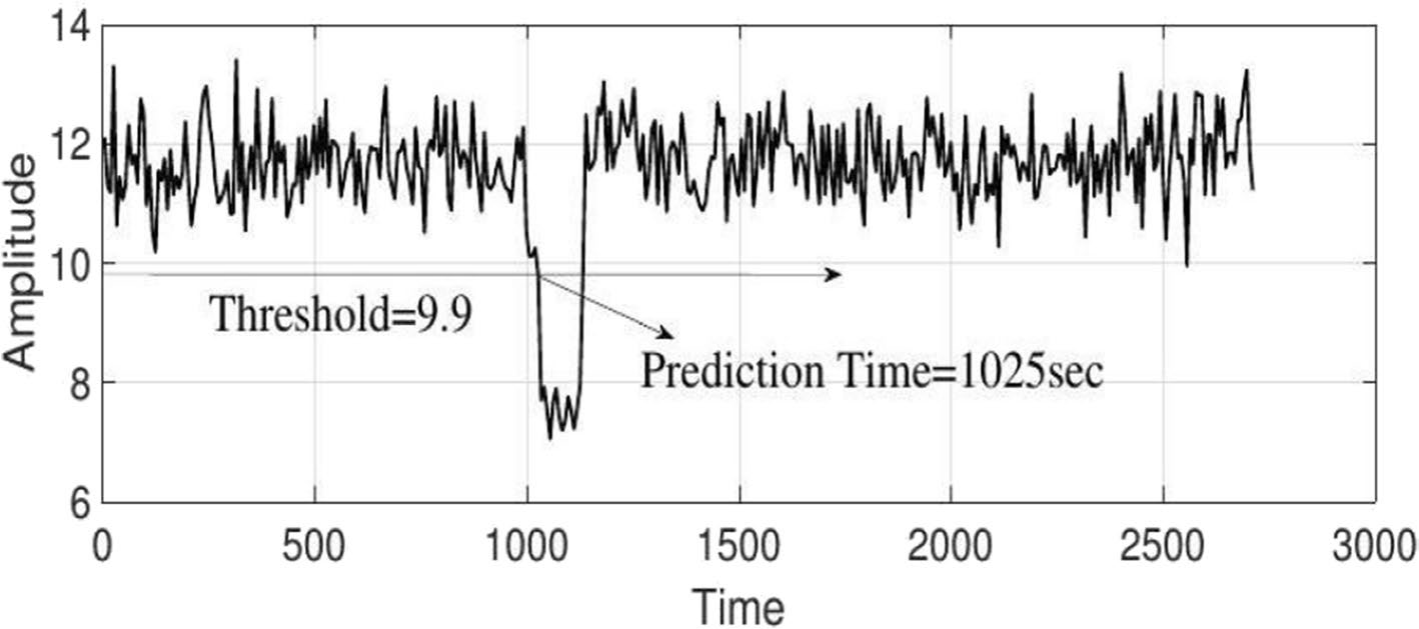
Output prediction with the proposed method on the adult epileptic model (start of IT and ictal periods at 1000 and1030sec, respectively)

To evaluate the performance of the algorithm with other studies, we use the criteria of sensitivity, specificity, accuracy, and FPR, which are respectively obtained (15), (16), (17), and (18) [28].

15 $$\text{Accuracy }=\frac{TP+TN}{TP+TN+FP+FN}$$

16 $$\text{Sensitivity }=\frac{TP}{TP+FN}$$

17 $$\text{Specificity }=\frac{TN}{TN+FP}$$

18 $${\text{FPR}}=\frac{FP}{\text{ whole of Interictal hours}}$$

where $TN,TP,FN$ and $FP$ are the true negative, true positive, false negative and false positive respectively. To $TN$ and $FP$, it is necessary to segment the EEG signal with the SOP length. Table 2 presents the evaluation outcomes of the proposed algorithm. The prediction results were obtained with a sensitivity of 87.5%, specificity of 95.6%, an accuracy of 92.6%, and an FPR of 1.50 with an average prediction time of 18.5 s. The prediction time, depending on the duration of the patient’s response to the alert of at least 10 s before the onset of a seizure is also perusing, which will average 31 s. Also, one of the outputs of the proposed adult model which includes interictal and IT periods is shown in Fig. 8 and indicates the prediction ability of the proposed method by setting the appropriate threshold. It should be noted that in patients whose ictal test data are not available, the thresholds were obtained based on ictal and interictal training data and their specificity and FPR criterias were evaluated.

**Table 2:** Results obtained from the proposed prediction method

Pat. n	Sens%	Spec%	Acc%	FPR	Ave.Pre Times (sec)
$1$	-	100	-	0	-
$2$	75	88.3	86.9	4.19	41
$3$	100	98.4	98.5	0.5	5
$4$	100	100	100	0	20
$5$	-	100	-	0	-
$6$	-	100	-	0	-
$7$	100	100	100	0	27
$8$	50	88.4	86.3	3.9	2
$9$	-	100	-	0	-
$10$	100	81.4	84.3	6.6	10
Ave	$87.5$	$95.6$	$92.6$	$1.50$	$18.5$

*Sens* sensitivity, *Spec* specitivity, *Acc* accuracy, *Ave.Pre Time* Average of prediction times, *Ave* Average

### Performance comparison

A few studies have been performed to predict short-term seizures. As a comparison between our proposed method and other exciting methods, we applied ref [18]’s and [20]’s methods on our existing EEG data, also the average results are given in Table 8. The results of [18]’s method previously described are shown in Table 3. We weighed up our method with the [20]’ threshold-based classification from among three classification algorithms presented in it. In [20], the postictal is presumed to be 1000 s while 150 s is set for it.

**Table 3:** Results obtained from the reference method [18]

Pat. n	Sens %	Spec%	Acc%	FPR	Ave.Pre Times (sec)
$1$	40.4	-	-	21.2	-
$2$	100	76.5	78.7	8.43	38
$3$	100	93.3	93.5	2.4	2
$4$	100	70	71	10.7	50.5
$5$	-	90.8	-	3.2	-
$6$	-	100	-	0	-
$7$	100	28.9	31	10.4	36.5
$8$	0	100	92.4	0	
$9$	-	42.8	-	20.5	-
$10$	50	53.8	53.3	16.6	1.5
Ave	$75$	$69.7$	$70$	$9.3$	$26.2$

due to the length limit of the available dataset. The number of patients is 60% of the total number of patients, the training seizure instances is obtained by randomly selecting [0.5 total number of seizure instances] for every patient, so four patients are removed from the dataset, the details of patients’ EEG are given in Table 4. All the results are average over the q runs which is recorded in the table. Since two sets of WPLI and PLI features in three different lengths T1, T2, and T3 are equal to 150, 200, and 300 respectively. Here, the average ictal period is considered to be 70 s, so the SOP lengths are achieved at 370, 420, and 520 respectively (for T1: average ictal length + T1 + postictal = 370). Table 5 and Table 6 show the results of WPLI and PLI features for T1 and T2 respectively, in which WPLI outcomes indicate higher average accuracy and the lower average FPR than PLI. WPLI, PLI-T3 in Table 7, the average accuracy rate of WPLI is inferior than that of PLI but its FPR is better. Table 8 also includes the best performance of [20] on the available dataset. Also, according to the results, it can be understood that the sensitivity, specificity, accuracy, and FPR of the proposed method are superior to the criteria [18] and [20]. The accuracy, FPR, and prediction time of the proposed method are 92.6%, 1.5, and 18.5 s, respectively. Significant high and low values in accuracy and FPR can be observed in our method, respectively. Our prediction time is proper for alleviating life threatening events and initial management of epileptic seizures before clinical symptoms and the false alarm rate is the lowest.

**Table 4:** Information of patients' EEG data employed in [20]'s method

Pat.$\mathbf{n}$	#Sei Tr	#Sei.Te	Int (sec)	Ict $(\mathbf{s}\mathbf{e}\mathbf{c})$	$\mathbf{q}$
$2$	3	3	5503	273	5
$3$	3	2	8612	376	5
$4$	1	1	5207	221	2
$7$	1	1	4605	172	2
$8$	2	2	6008	416	5
$10$	2	1	3520	300	3

${\text{q}}=$ #runs, Int = Interictal, Ict = Ictal

**Table 5:** WPLI-T1 and PLI-T1 results obtained from the [20]'s method

	WPLI-T1							**PLI- T1**		
**Pat. n**	**Sens%**	**Spec%**	**Acc%**	**FPR**	Ave.Pre Times (sec)	**Sens%**	**Spec%**	**Acc%**	**FPR**	PreTime (sec)
$2$	28.6	37.6	30.9	4.28	78	20.9	19.3	19.8	5.6	27
$3$	0	14.39	18.88	7.11	-	14.3	17.5	27.4	6.6	99
$4$	50	77	77	1.75	82	50	77	77	1.75	82
$7$	71	41	42.8	6.4	344	71	41	42.8	6.4	344
$8$	2.2	12.2	4.4	3.4	101	2.2	9.6	7.6	3.7	101
$10$	0	100	74.5	0	-	0	26.4	62.7	1.29	-
Ave	$25.3$	$47$	$41.4$	$3.82$	$120$	$26.4$	$31.8$	$39.55$	$4.22$	$101$

Pat.${\text{n}}=$Patients number, Sens$=$sensitivity,${\text{Spec}}=$specitivity,${\text{Acc}}=$accuracy, PreTime$=$prediction time

**Table 6:** WPLI-T2 and PLI-T2 results obtained from the [20]'s method

	WPLI- T2						PLI- T2			
**Pat.** $\mathbf{n}$	**Sens%**	**Spec%**	**Acc%**	**FPR**	Ave.Pre Times (sec)	**Sens%**	**Spec%**	**Acc%**	**FPR**	PreTime (sec)
$2$	22.4	31.9	26.7	4.77	103	22.4	33.2	26.7	5.12	103
$3$	96.3	19.6	40.4	6.1	61	6.2	36.9	46.4	5.4	129
$4$	50	75	75	1.84	82	50	75	75	1.84	82
$7$	0	19	16	7.46	-	0	19	16	7.46	-
$8$	12	21.2	17.4	6.4	3	27.5	15.2	16.2	6.7	75
$10$	0	58.8	44.1	3	-	0	58.8	44.1	3.1	-
Ave	$30$	$37.5$	$36.6$	$4.92$	$71.4$	$17.6$	$39.6$	$10$	$4.93$	$96$

**Table 7:** WPLI-T3 and PLI-T3 results obtained from the [20]'s method

	WPLI-T3					PLI- T3				
Pat. n	Sens%	Spec%	Acc%	FPR	Ave.Pre Times (sec)	Sens%	Spec%	Acc%	FPR	PreTime (sec)
$2$	61.8	44.3	51.4	2.83	204	69.6	44.3	51.41	3.72	199
$3$	40.1	9.7	22.9	5.6	155	42.1	23.5	36.1	5.2	132
$4$	0	71	66	2.14	-	0	71	66	2.14	-
$7$	100	64	69	2.71	264	100	64	69	2.71	264
$8$	31.1	26.3	23.7	4.8	202	35.3	26.3	23.7	4.9	209
$10$	0	29.4	19.6	3.1	-	0	29.4	19.6	3.1	-
Ave	$38.8$	$40.7$	$42$	$3.53$	$199$	$41$	$43$	$44$	$3.6$	$193$

**Table 8:** Comparison with other seizure prediction methods applied to our existed dataset

	Sens%	Spec%	Acc%	FPR	PreTime (sec)
Ave’ [18]	75	69.7	70	9.3	26.2
Best Ave' $\left[20\right]$	58.5	55	56	3.19	$173.8$
Our Ave	$87.5$	$95.6$	$92.6$	1.50	18.52

## Conclusion

In this study, a short-term seizure prediction algorithm was presented in patients with temporal lobe epilepsy based on AR-Cepstrum Analysis and Signal Morphology. To this, goal, an algorithm was proposed based on three aims: 1) IT detection with AR model and Cepstrum analysis, 2) DWT and median filter steps to increase accuracy and reduce artifact according to signal morphology in periods and channels, and 3) averaging and positive envelope steps to determine the patient-specific thresholds.

In this method, we tracked the changes in the behavior of the epileptic focus area in two opposite recording channels in the time domain. To simplify the algorithm, the use of two channels and the first coefficient of Cepstrum was considered the most effective coefficient and the thresholding method instead of classification. Another advantage of the algorithm is the ability to reduce artifacts that are in different frequencies, which are separated by a wavelet transform. Because two channel artifacts are unequal at the same time and in the same channel at different times, artifacts are reduced by comparing the channels to each other and the other one. Our experimental results and the comparison with other works illustrate that the proposed method is efficient, reliable, and suitable for short-term seizure prediction. This is by reaching accuracy higher than other methods with proper prediction time to initial management of epileptic seizures before clinical symptoms and alleviate risks of seizure. For more reliability, the performance of the algorithm was evaluated on the adult epileptic EEG model and yielded acceptable results. This algorithm can be developed in portable alarm devices for real-life use and patient-specific non-invasive data with artifacts. It is recommended to test the algorithm on more patients to confirm its performance clinically.

## Data Availability

The datasets generated during and/or analysed during the current study are not publicly available due [by obtaining permission from Medical Sciences of Kerman University, the EEG data of epileptic patients were provided to us, however, there is no permission to publish the data] but are available from the corresponding author on reasonable request.

## References

[1] Engelborghs S, Dhooge R, De Deyn P (2000). Pathophysiology of epilepsy. Acta Neurol Belg.

[2] Alickovic E, Kevric J, Subasi A (2018). Performance evaluation of empirical mode decomposition, discrete wavelet transform, and wavelet packed decomposition for automated epileptic seizure detection and prediction. Biomed Signal Process Control.

[3] Assi EB, Nguyen DK, Rihana S, Sawan M (2017). Towards accurate prediction of epileptic seizures: A review. Biomed Signal Process Control.

[4] Luders HO. The ictal onset zone, in Textbook of epilepsy surgery. United Kingdom: CRC Press; 2008. p. 681–701.

[5] Mousavi S, Niknazar M, Vahdat BV, “Epileptic seizure detection using ar model on eeg signals”, in,. (2008). Cairo International Biomedical Engineering Conference. IEEE.

[6] Chisci L, Mavino A, Perferi G, Sciandrone M, Anile C, Colicchio G, Fuggetta F (2010). Real-time epileptic seizure prediction using ar models and support vector machines. IEEE Trans Biomed Eng.

[7] Mohamadi S, Amindavar H, Hosseini SAT. Arima-garch modeling for epileptic seizure prediction, in 2017 IEEE International Conference on Acoustics, Speech and Signal Processing (ICASSP). IEEE. 2017;994–998.

[8] M. Bozek-Juzmicki, D. Colella, and G. M. Jacyna, “Feature-based epileptic seizure detection and prediction from ecog recordings,” in Proceedings of IEEE-SP International Symposium on Time-Frequency and Time-Scale Analysis. IEEE, 1994, pp. 564–567.

[9] Yu P-N, Naiini SA, Heck CN, Liu CY, Song D, Berger TW, “A sparse laguerre-volterra autoregressive model for seizure prediction in temporal lobe epilepsy”, in, (2016). 38th Annual International Conference of the IEEE Engineering in Medicine and Biology Society (EMBC). IEEE.

[10] Winterhalder M, Schelter B, Maiwald T, Brandt A, Schad A, Schulze-Bonhage A, Timmer J (2006). Spatio-temporal patient– individual assessment of synchronization changes for epileptic seizure prediction. Clin Neurophysiol.

[11] L. Tong, W. Wang, N. Zhao, and X. Huang, “The method evaluation for preictal prediction of epilepsy with strong-noise eeg and simulation of automatic drug release system,” in 2010 3rd International Conference on Biomedical Engineering and Informatics, vol. 3. IEEE, 2010, pp. 1054–1058.

[12] Teixeira C, Direito B, Feldwisch-Drentrup H, Valderrama M, Costa R, Alvarado-Rojas C, Nikolopoulos S, Le Van Quyen M, Timmer J, Schelter B (2011). Epilab: A software package for studies on the 11 prediction of epileptic seizures. J Neurosci Methods.

[13] Kamath C (2014). Automatic seizure detection based on teager energy cep strum and pattern recognition neural networks. QScience Connect.

[14] Kamath CC. Comparison of baseline cepstral vector and composite vectors in the automatic seizure detection using probabilistic neural networks. Int Sch Res Notices. 2013;2013:1–9.

[15] Ren H, Qu J, Chai Y, Huang L, Tang Q (2018). Cepstrum coefficient analysis from low-frequency to high-frequency applied to automatic epileptic seizure detection with bio-electrical signals. Appl Sci.

[16] Gotman J, Gloor P (1976). Automatic recognition and quantification of interictal epileptic activity in the human scalp eeg. Electroencephalog raphy and clinical neurophysiology.

[17] Gotman J (1982). Automatic recognition of epileptic seizures in the eeg. Electroencephalogr Clin Neurophysiol.

[18] Osorio I, Frei MG, Wilkinson SB (1998). Real-time automated detection and quantitative analysis of seizures and short-term prediction of clinical onset. Epilepsia.

[19] Daoud HG, Abdelhameed AM, Bayoumi M, “Automatic epileptic seizure detection based on empirical mode decomposition and deep neural network”, in, (2018). IEEE 14th International Colloquium on Signal Processing & Its Applications (CSPA). IEEE.

[20] Detti P, de Lara GZM, Bruni R, Pranzo M, Sarnari F, Vatti G (2018). A patient-specific approach for short-term epileptic seizures prediction through the analysis of eeg synchronization. IEEE Trans Biomed Eng.

[21] R. C. Gonzalez, Digital image processing. Pearson education india, 2009.

[22] S. S. Haykin, Adaptive filter theory. Pearson Education India, 2008.

[23] Sderstrm T, Stoica P. Model validation and model structure determination, in system identification. Prentice-Hall International. 1989;422–467.

[24] L. R. Rabiner and R. W. Schafer, Introduction to digital speech process ing. Now Publishers Inc, 2007, vol. 1.

[25] Celka P, Colditz P (2002). Nonlinear nonstationary wiener model of infant eeg seizures. IEEE Trans Biomed Eng.

[26] Roessgen M, Zoubir AM, Boashash B (1998). Seizure detection of newborn eeg using a model-based approach. IEEE Trans Biomed Eng.

[27] Lopes da Silva F, Hoeks A, Smits H, Zetterberg L (1974). Model of brain rhythmic activity. the alpha-rhythm of the thalamus. Kybernetik.

[28] H. Alawieh, H. Hammoud, M. Haidar, M. H. Nassralla, A. M. El Hajj, and Z. Dawy, “Patient-aware adaptive ngram-based algorithm for epileptic seizure prediction using eeg signals,” in 2016 IEEE 18th International Conference on e-Health Networking, Applications and Services (Healthcom). IEEE, 2016, pp. 1–6.

